# Country Seat

**Published:** 1869-05

**Authors:** 


### COUNTRY SEAT.

One of the most convenient, elegant, imposing and roomy family mansions in New England is for lease or sale, with all the furniture, horses, carriages, sleighs, &c., &c. Immediate possession, as the owner is going abroad. If purchased, almost the entire purchase-money may remain on long interest, at six per cent.; or it will be exchanged for productive city property in New York, Philadelphia, or Boston. It is two hours by rail from Boston, or Newport, Rhode Island. The whole place, with fourteen acres, is in perfect order, inside and out, and can be purchased for one half the cost of the buildings alone, just completed before the war. Such an opportunity is seldom presented. Photographs at the Editor's office, 176 Broadway, New York.

